# Alterations of epithelial stem cell marker patterns in human diabetic corneas and effects of *c-met* gene therapy

**Published:** 2011-08-12

**Authors:** Mehrnoosh Saghizadeh, Siavash Soleymani, Angel Harounian, Bhavik Bhakta, Sergey M. Troyanovsky, William J. Brunken, Graziella Pellegrini, Alexander V. Ljubimov

**Affiliations:** 1 Ophthalmology Research Laboratories, Cedars-Sinai Medical Center, Los Angeles, CA; 2 University of California Los Angeles, Los Angeles, CA; 3 Department of Dermatology, Northwestern University, Chicago, IL; 4 Departments of Ophthalmology and Cell Biology, State University of New York, Downstate Medical Center, Brooklyn, New York, NY; 5 Centre for Regenerative Medicine, University of Modena e Reggio Emilia, Modena, Italy

## Abstract

**Purpose:**

We have previously identified specific epithelial proteins with altered expression in human diabetic central corneas. Decreased hepatocyte growth factor receptor (c-met) and increased proteinases were functionally implicated in the changes of these proteins in diabetes. The present study examined whether limbal stem cell marker patterns were altered in diabetic corneas and whether *c-met* gene overexpression could normalize these patterns.

**Methods:**

Cryostat sections of 28 ex vivo and 26 organ-cultured autopsy human normal and diabetic corneas were examined by immunohistochemistry using antibodies to putative limbal stem cell markers including ATP-binding cassette sub-family G member 2 (ABCG2), N-cadherin, ΔNp63α, tenascin-C, laminin γ3 chain, keratins (K) K15, K17, K19, β_1_ integrin, vimentin, frizzled 7, and fibronectin. Organ-cultured diabetic corneas were studied upon transduction with adenovirus harboring *c-met* gene.

**Results:**

Immunostaining for ABCG2, N-cadherin, ΔNp63α, K15, K17, K19, and β_1_ integrin, was significantly decreased in the stem cell-harboring diabetic limbal basal epithelium either by intensity or the number of positive cells. Basement membrane components, laminin γ3 chain, and fibronectin (but not tenascin-C) also showed a significant reduction in the ex vivo diabetic limbus. *c-Met* gene transduction, which normalizes diabetic marker expression and epithelial wound healing, was accompanied by increased limbal epithelial staining for K17, K19, ΔNp63α, and a diabetic marker α_3_β_1_ integrin, compared to vector-transduced corneas.

**Conclusions:**

The data suggest that limbal stem cell compartment is altered in long-term diabetes. Gene therapy, such as with c-met overexpression, could be able to restore normal function to diabetic corneal epithelial stem cells.

## Introduction

In pathological conditions, such as diabetes mellitus, the cornea is significantly affected and this can cause visual impairment. The most recognized diabetic complications in the cornea include neurotrophic corneal ulcers, filamentous keratitis, loss of corneal sensation, and a characteristic epithelial keratodystrophy, which is referred to as diabetic keratopathy [1-9]. Diabetic cornea exhibits basement membrane abnormalities, reduced numbers of hemidesmosomes, altered growth factor content and signaling, epithelial cellular enlargement, edema, and delayed wound healing resulting in persistent epithelial defects [2-4,8-11]. Treatment for diabetic keratopathy remains symptomatic [2].

Corneal epithelial renewal and healing of epithelial wounds largely depend on corneal stem cells that, at least in humans, reside in the basal epithelial layer of the corneoscleral junction, limbus [12-21]. These cells represent less than 10% of the total limbal basal epithelial cell population [22,23]. Deficiencies of or damage to these limbal epithelial stem cells (LESC) have serious implications for corneal function such as in-growth of conjunctival cells and neovascularization of the corneal stroma, which eventually lead to corneal opacity and vision loss [20,24-26]. These cells have a high capacity for self-renewal, which is retained throughout life. Corneal maintenance depends on LESC as a source of epithelial proliferation and rapid renewal through generation of transient amplifying (TA) cells, which in turn differentiate into epithelial cells during their centripetal movement [21,27-29].

Because of its role in epithelial renewal and wound healing, deficiency of the limbal niche and its residing LESC may be responsible for abnormalities in diabetic corneal epithelium. In the present paper we examined various putative stem cell markers in ex vivo diabetic and normal epithelial limbal compartment, as well as in organ-cultured diabetic corneas upon overexpression of *c-met* proto-oncogene shown to normalize wound healing time and epithelial marker expression [30]. Immunostaining patterns of several putative stem cell markers were altered in the diabetic limbus, and some of these patterns could be normalized by c-met overexpression. The data suggest that limbal compartment may play an important role in diabetic corneal alterations that can be corrected by gene therapy.

## Methods

### Tissues

Age-matched normal, diabetic (with insulin-dependent [IDDM] or non-insulin-dependent [NIDDM] diabetes), and diabetic retinopathy (DR) autopsy human corneas were obtained from the National Disease Research Interchange (NDRI, Philadelphia, PA), within 24 (for ex vivo) to 48 h after death. NDRI has a human tissue collection protocol approved by a managerial committee and subject to National Institutes of Health oversight. In this study (Table 1), 15 normal (from 13 donors, mean age 57.8±21.8 years) and 13 diabetic (from 9 donors; mean age 71.2±6.3 years; 7 with IDDM, 2 with NIDDM, 4 with DR) ex vivo corneas, as well as 13 pairs of organ-cultured diabetic corneas (from 13 donors; mean age 68.5±14.4 years; 6 with IDDM, 7 with NIDDM, 4 with DR) were used. Mean ages in all groups as well as mean disease durations for known cases in ex vivo and organ culture diabetic groups did not differ significantly. The corneas were embedded in Optimal Cutting Temperature (OCT) compound (Sakura Finetek USA, Inc., Torrance, CA) and stored at –80 °C for immunohistochemistry, or were processed for organ culture.

**Table 1:** Donor characteristics.

**Case number**	**Diabetes type**	**Age, sex**	**Diabetes duration, years**	**Cause of death**
**Ex vivo normal**				
95–12	-	17, F	-	gunshot wound
95–15	-	59, M	-	cardiac arrest
95–44	-	68, M	-	massive hemorrhage
99–19	-	79, F	-	respiratory arrest
99–61	-	80, F	-	myocardial infarction
03–3	-	71, M	-	myocardial infarction
03–09	-	72, M	-	lung cancer
05–16	-	56, M	-	ruptured aortic dissection
05–25	-	60, F	-	COPD
05–26	-	60, F	-	COPD
05–45	-	10, M	-	exsanguination
05–46	-	10, M	-	exsanguination
05–56	-	45, M		cardiovascular accident
05–60	-	65, F	-	pneumonia
10–03	-	69, M	-	respiratory failure
**Ex vivo diabetic**				
95–17	IDDM	79, M	5	cardiac arrest
95–18	IDDM	79, M	5	cardiac arrest
96–06	IDDM	69, M	22	acute cardiac event
96–30	NIDDM	77, F	>5	cardiorespiratory arrest
96–46	IDDM, PDR	68, F	>48	cardiorespiratory arrest
96–47	IDDM, PDR	68, F	>48	cardiorespiratory arrest
96–95	IDDM	63, M	3	cardiovascular accident
96–96	IDDM	63, M	3	cardiovascular accident
99–08	IDDM, DR	67, F	30	myocardial infarction
99–79	IDDM, PDR	77, F	unknown	cardiac arrest
99–80	IDDM, PDR	77, F	unknown	cardiac arrest
01–47	IDDM, DR	64, F	unknown	ventricular arrhythmia
06–26	NIDDM	77, M	15	intracerebral hemorrhage
**Organ-cultured diabetic**				
07–27	IDDM	81, M	>10	acute renal failure
07–32	NIDDM	84, M	20	stroke
07–34	IDDM, DR	37, M	22	intracranial hemorrhage
08–35	IDDM, DR	88, M	38	cardiac arrest
08–36	NIDDM	82, M	15	stroke
08–38	IDDM	73 F	>10	diabetic ketoacidosis
08–40	NIDDM	59, M	20	cardiac arrest
08–44	IDDM, DR	71, M	15	cardiopulmonary arrest
08–49	NIDDM	59, F	28	intracranial hemorrhage
08–54	NIDDM	57, M	10	myocardial infarction
08–57	IDDM	78, F	15	respiratory failure
09–12	NIDDM, DR	61, F	unknown	myocardial infarction
09–16	NIDDM	61, F	>10	congestive heart failure

M, male; F, female; PDR, proliferative diabetic retinopathy; COPD, chronic obstructive pulmonary disease. In the ex vivo groups, each case number refers to one cornea; in organ-cultured diabetic group, each case number refers to a pair of fellow corneas.

### Corneal organ culture and viral transduction

As described previously [30,31], after filling the corneal concavity with warm agar-collagen mixture, corneas were cultured in serum-free medium with insulin-transferrin-selenite, antibiotics and antimycotic (Invitrogen, Carlsbad, CA), at a liquid-air interface with epithelium facing upwards. Organ-cultured diabetic corneas were transduced for 48 h with 1.0–2.0×10^8^ plaque-forming units of recombinant adenoviruses, rAV-cmet (harboring full-length *c-met* open reading frame) and the fellow corneas with rAV-vector (no gene inserted) as a control. Seventy-five μg/ml of sterile sildenafil citrate (Viagra®; Pfizer Corp., New York, NY) was added to the culture medium along with the viruses to increase rAV transduction efficiency [31]. Some transduced corneas were processed after 7–10 days in culture, some after wound healing experiments [30]. They were embedded in OCT and 5 μm cryostat sections cut for immunostaining on a Leica CM1850 cryostat (McBain Instruments, Chatsworth, CA).

### Immunohistochemistry

The list of primary antibodies to putative stem cell markers is presented in Table 2. Different fixations such as 100% acetone at –20 °C for 10 min, 100% methanol at –20 °C for 10 min, 1% formalin (0.37% formaldehyde) in saline at room temperature for 5 min were used for different antibodies. For each marker the same exposure time was used when photographing stained sections of fellow corneas using a MicroFire digital camera (Optronics, Goleta, CA) attached to an Olympus BX40 microscope (Olympus USA, Melville, NY) and operated using PictureFrame software. Negative controls without a primary antibody were included in each experiment.

**Table 2:** Antibodies used in the study.

**Antigen**	**Antibody**	**Source**	**Dilution**	**Immunostaining**
ABCG2	Mouse mAb MAB4155	Millipore	1:50	-
ABCG2	Mouse mAb sc-58222	Santa Cruz Biotechnology	1:5	+
ABCG2	Rabbit pAb sc-25821	Santa Cruz Biotechnology	1:20	+
C/EBPδ	Rabbit pAb sc-636	Santa Cruz Biotechnology	1:20	-
Fibronectin	Mouse mAb 568	[72]	1:60	+
Integrin β1	Mouse mAb MAB1959	Millipore	1:50	+
Integrin β1	Mouse mAb MAB2000	Millipore	1:50	+
Keratin 15	Mouse mAb sc-47697	Santa Cruz Biotechnology	1:10	+
Keratin 17	Mouse mAb E3	[73]	straight	+
Keratin 17	Rabbit mAb #4543	Cell Signaling	1:50	+
Keratin 19	Mouse mAb MAB1607	Millipore	1:10	+
Keratin 19	Mouse mAb MA1–35554	Thermo Scientific	1:10	+
Keratin 19	Rabbit pAb PA1–38014	Thermo Scientific	1:20	+
Keratin 19	Mouse mAb MAB1608	Millipore	1:10	+
Laminin β1	Rat mAb LT3	[74]	straight	+
Laminin β2	Mouse mAb C4	Developmental Hybridoma Bank	straight	+
Laminin γ1	Rat mAb A5	[75]	straight	+
Laminin γ3	Rabbit pAb R96	[76]	1:500	+
Laminin γ3	Rabbit pAb sc-25719	Santa Cruz Biotechnology	1:20	-
Laminin γ3	Goat pAb sc-16601	Santa Cruz Biotechnology	1:10	+
N-Cadherin	Mouse mAb 3B9	Invitrogen	1:20	+
N-Cadherin	Rabbit pAb 12221	Abcam	1:50	-
N-Cadherin	Rabbit pAb sc-7939	Santa Cruz Biotechnology	1:20	+
Nidogen-1	Mouse mAb MAB2570	R&D Systems	1:50	+
Nidogen-2	Rabbit pAb 1080	[77]	1:200	+
Nidogen-2	Goat pAb sc-26132	Santa Cruz Biotechnology	1:25	-
Nidogen-2	Goat pAb sc-26133	Santa Cruz Biotechnology	1:25	-
ΔNp63	Goat pAb sc-8609	Santa Cruz Biotechnology	1:20	+
ΔNp63	Rabbit pAb 619001	Biolegend	1:50	-
P63α	Rabbit pAb	[23]	1:100	+
SOD2	Rabbit pAb sc-30080	Santa Cruz Biotechnology	1:10	+
Vimentin	Goat pAb sc-7558	Santa Cruz Biotechnology	1:20	+
Frizzled 7	Rat mAb 1981	R&D Systems	1:20	+
Tenascin-C	Mouse mAb BC2	[78]	straight	+

mAb, monoclonal antibody; pAb, polyclonal antibody.

### Statistics

Immunostaining results were analyzed by unpaired (for ex vivo corneas; n=15 for normal, n=13 for diabetic) or paired (for organ cultured corneas; n=13 pairs) Student’s *t*-test (InStat, GraphPad Software, San Diego, CA). Staining intensity was scored arbitrarily as: 0 (negative), 0.5 (weak), 1 (distinct), 2 (moderate), 3 (strong), 4 (very strong). Most cases were stained at least twice with good reproducibility between experiments, and a mean intensity score from independent experiments was used for each case. The mean scores between groups (e.g., normal versus diabetic) were then compared. A p-value <0.05 was considered significant. Data are expressed as mean±standard error (SEM).

## Results

### Distribution of putative stem cell markers in normal and diabetic ex vivo corneas

**Figure 1:**
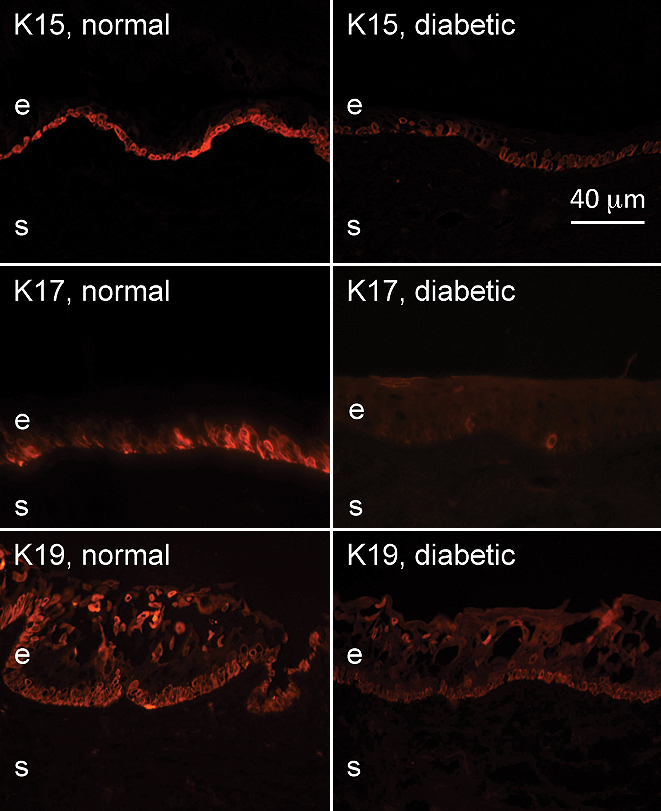
Keratin expression patterns in normal and diabetic ex vivo limbus. The staining intensity of K15, K17, and K19 was significantly decreased in the diabetic limbus. Note a reduction of K17-positive cells in the diabetic limbus as well. Here and in all other figures, each normal and diabetic pair was photographed at the same exposure times in the same staining experiments. e, epithelium, s, stroma. Bar=40 μm.

**Figure 2:**
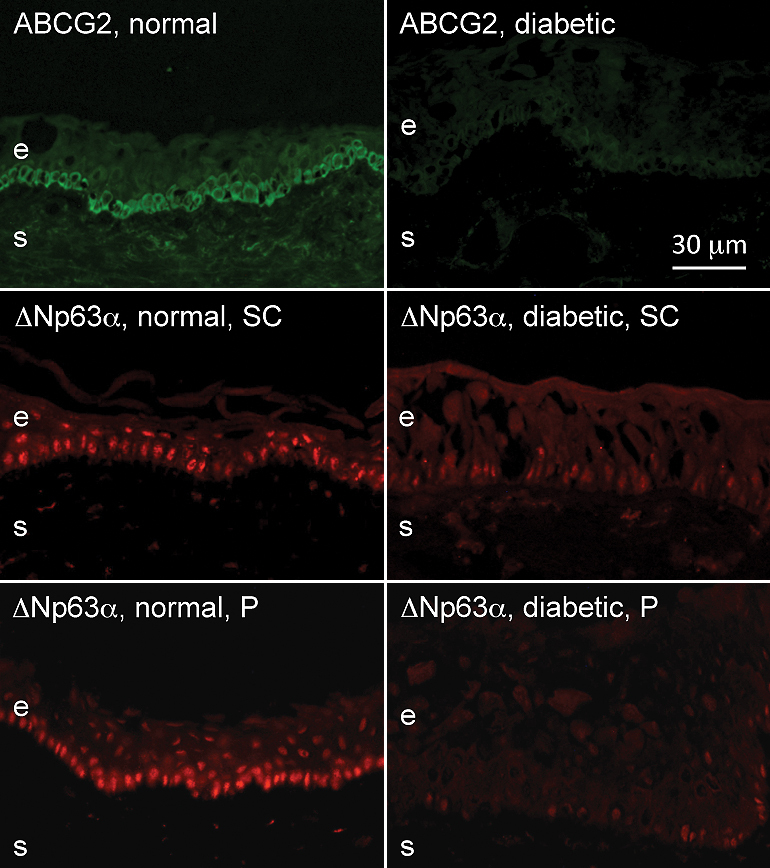
Putative LESC marker expression patterns in normal and diabetic ex vivo limbus. Note a dramatic decrease in staining intensity and the number of positive basal epithelial cells for ABCG2 and ΔNp63α in the diabetic limbus. ΔNp63α was revealed with two different antibodies (Santa Cruz, SC) and Pellegrini (P) with the same result. e, epithelium, s, stroma. Bar=30 μm.

**Figure 3:**
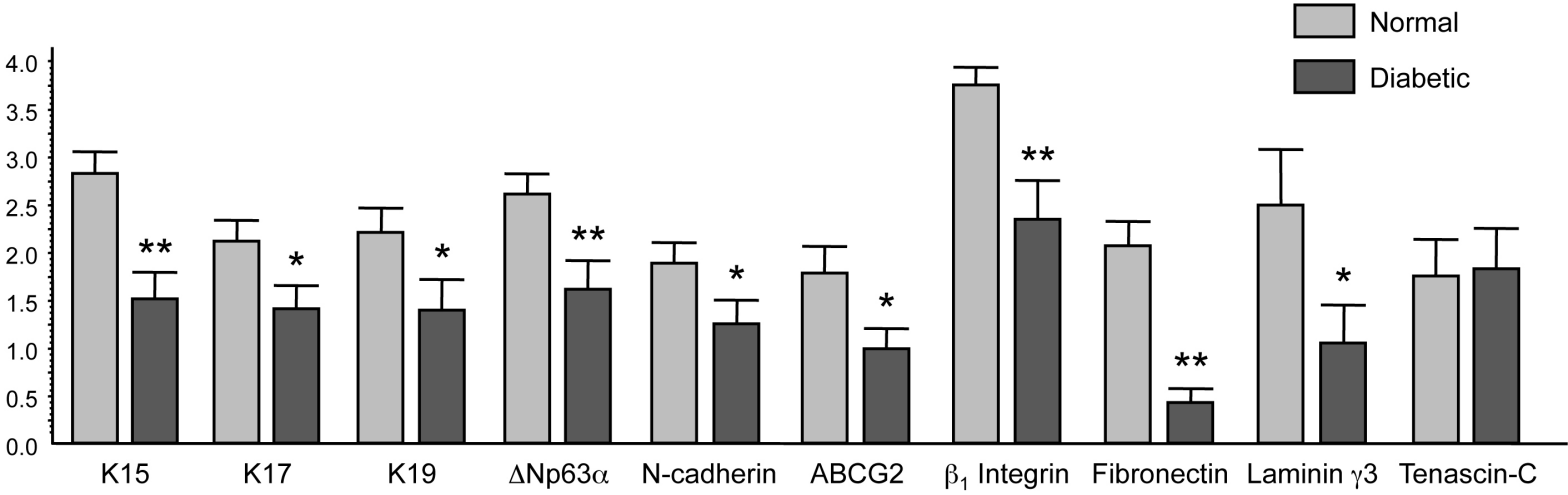
Statistical analysis of changes in the staining for various markers in diabetic versus normal ex vivo limbus. Significant staining decrease was observed for K15, K17, K19, ΔNp63α, N-cadherin, ABCG2, fibronectin, β_1_ integrin, and laminin γ3 chain. Data are mean±SEM. Normal, n=15; diabetic, n=13. *p<0.05; **p<0.01. Details are in the Methods section.

**Figure 4:**
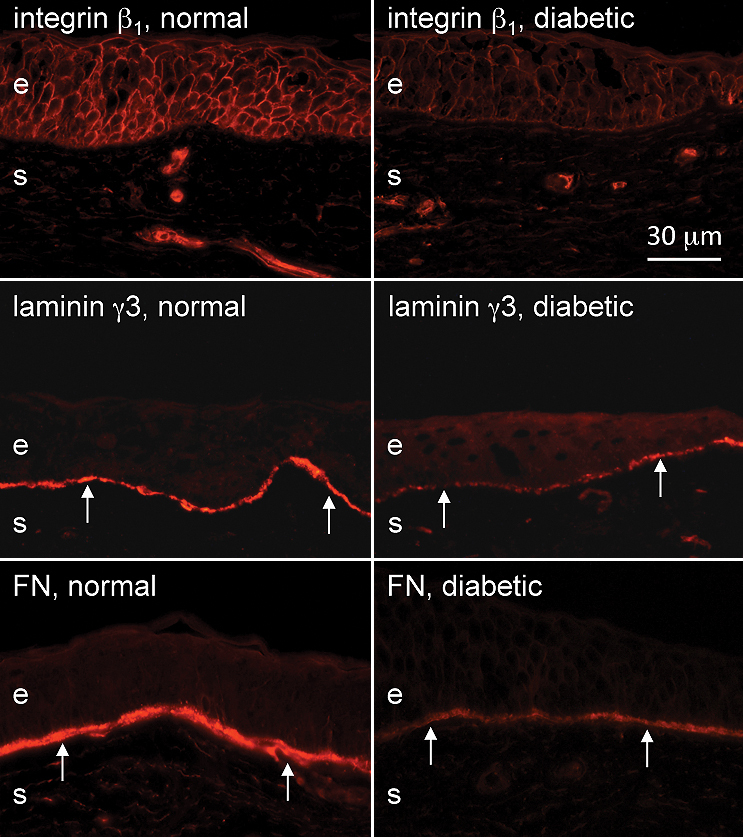
Integrin and basement membrane protein expression patterns in normal and diabetic ex vivo limbus. Integrin β_1_ staining is markedly reduced in the diabetic limbus, which occurs in all epithelial layers. A limbal-specific laminin γ3 chain staining is weak and discontinuous in the diabetic limbal epithelial basement membrane (arrows). This is also true for fibronectin. e, epithelium, s, stroma. Bar=30 μm.

The immunostaining patterns of several limbal and/or putative stem cell markers were altered in the ex vivo diabetic limbus compared to the normal one. Figure 1, left column, shows the normal staining patterns of cytoskeletal structural proteins of epithelial cells, keratins 15, 17, and 19 (K15, K17 and K19). These three keratins were expressed in limbal compartment but not in central ex vivo corneal epithelium, in good agreement with previous data [17,32-34]. K15 and K19 were prominently expressed in the basal limbal cells and in lesser amounts in the suprabasal and superficial layers of the limbal epithelium (Figure 1). K17 protein was usually found in clusters of limbal basal cells (Figure 1), as was vimentin (not shown here). As shown in Figure 1, right column, in the diabetic limbus staining for all three keratins decreased by both intensity and number of positive cells; the latter was most pronounced for K17. Decreased staining for these keratins in the diabetic limbus reached significance. The most commonly used putative LESC markers [20,23,35-37], ATP-binding cassette transporter Bcrp1/ABCG2 and transcription factor ΔNp63α isoform, were also significantly decreased in the diabetic limbus (Figure 2 and Figure 3). The same was true for another putative LESC marker [38], N-cadherin (Figure 3). β_1_ Integrin [39] that is found in basal cells as well as the suprabasal and superficial layers of the corneal epithelium was expressed much less in the diabetic limbus (Figure 4), although its localization did not agree with the presence in LESC niche only. In the diabetic limbus, reduced and discontinuous immunostaining was observed for select ECM markers, such as laminin γ3 chain (Figure 4), expressed mostly in the limbal basement membrane [40,41]. Fibronectin staining was also significantly decreased in the diabetic limbal basement membrane (Figure 4). No significant changes were observed in the diabetic limbal cells for total tenascin-C (Figure 3), a ubiquitous laminin γ1 chain, and putative LESC markers superoxide dismutase 2 (SOD2), vimentin, and a Wnt receptor, frizzled 7 (data not shown).

### Normalization of putative stem cell marker patterns upon c-met overexpression

**Figure 5:**
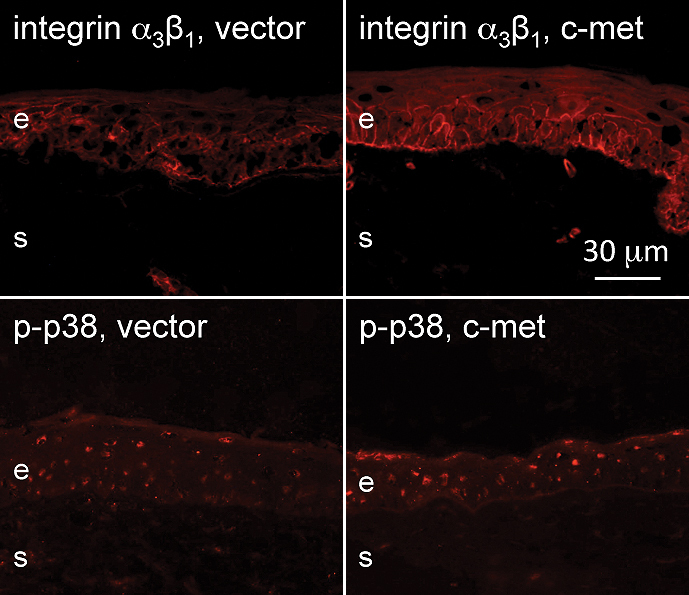
Increased diabetic marker expression in the diabetic limbus in organ culture upon c-met overexpression. Both integrin α_3_β_1_ and p-p38 staining in the limbal epithelium is increased upon *c-met* gene transduction and becomes similar to normal. e, epithelium, s, stroma. Bar=30 μm.

**Figure 6:**
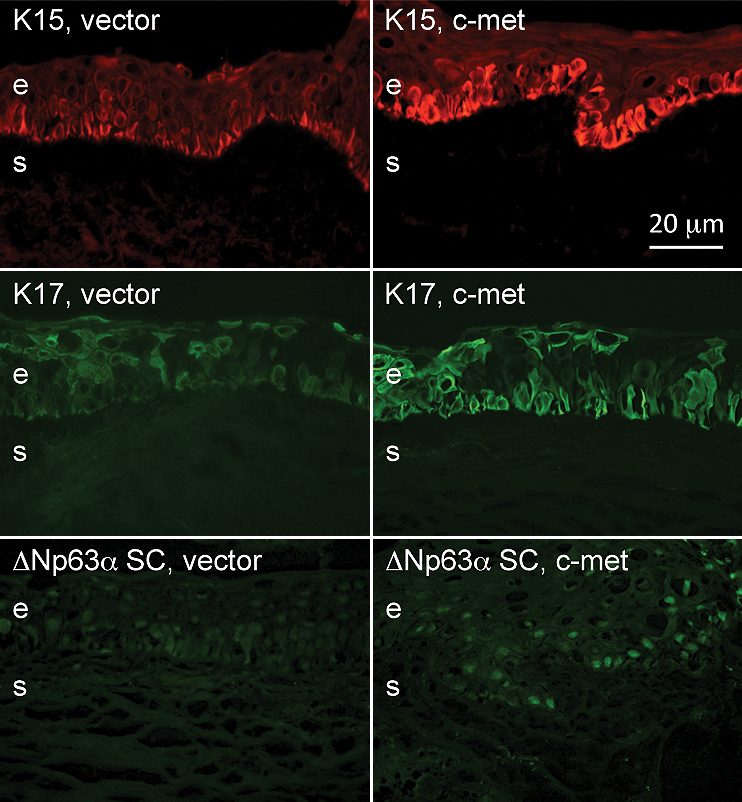
Increased putative LESC marker expression in the diabetic limbus in organ culture upon c-met overexpression. *c-Met* gene transduction leads to elevated expression of K15, K17, and ΔNp63α in the limbus of organ-cultured diabetic corneas. The staining intensity and regularity appear more normal (compare with Figure 1 and Figure 2). Note that in organ cultures keratins (especially K17) can also be seen in suprabasal epithelial layers. e, epithelium, s, stroma. Bar=20 μm.

**Figure 7:**
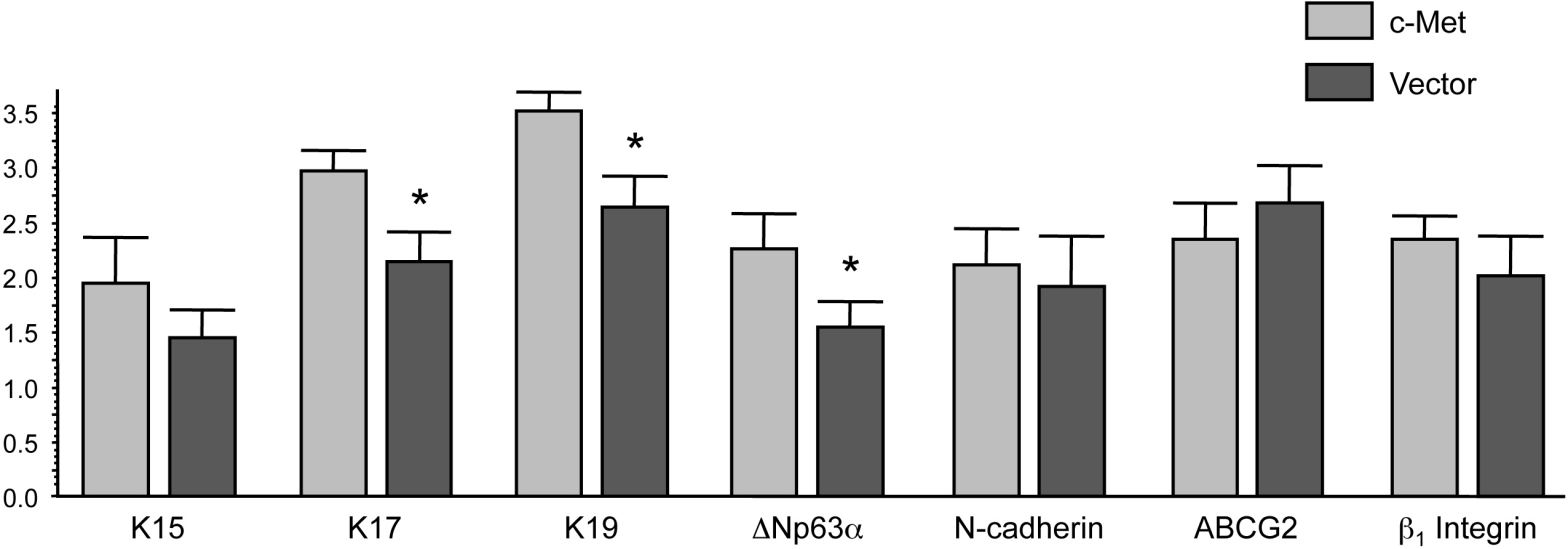
Statistical analysis of changes in the staining for various markers in the diabetic limbus in organ culture upon c-met overexpression. Significant staining increase after *c-met* gene transduction was observed for K17, K19, and ΔNp63α. Changes in the expression levels of K15, N-cadherin and β_1_ integrin did not reach significance. Data are mean±SEM. Thirteen pairs of c-met or vector treated organ cultured diabetic corneas were used. *p<0.05. Details are in the Methods section.

We have recently shown that the expression of certain markers altered in the ex vivo diabetic corneas including integrin α_3_β_1_, some laminin chains, nidogen-1, nidogen-2, and phosphorylated p38 MAP kinase (p-p38) returned to almost normal patterns in the central region of diabetic organ-cultured corneas after c-met overexpression using rAV-driven c-met transduction [30]. As shown in Figure 5, c-met transduction brought about an increase in staining for some of these markers, such as integrin α_3_β_1_ and p-p38, so that they became closer to normal (see [30]) in the diabetic limbus. We next examined if altered expressions of putative LESC markers could also be normalized by rAV-cmet transduction of diabetic organ-cultured corneas. Indeed, c-met overexpression was accompanied by increased limbal staining for K15 (did not reach significance), K17, and K19, as well as ΔNp63α isoform compared to vector-transduced corneas (Figure 6; compare with Figure 1 and Figure 2), so that the staining became similar to normal corneas. Some of these increases were significant (Figure 7), although certain markers did not show an appreciable change in staining intensity. It should be mentioned that in organ-cultured diabetic corneas some K17 and K19 immunostaining could also be found in the suprabasal layers of the limbal epithelium as well as in the central cornea, whereas K15 was still expressed exclusively in the limbus.

## Discussion

As a systemic disease, diabetes has significant impact on all tissues. In the eye, the major vision-threatening effect is on retina (DR), whereas the other ocular parts are generally thought to be less affected [42,43]. However, more than a half of diabetics suffer from corneal problems related to neuropathy and epitheliopathy [44]. Various epithelial abnormalities are present in diabetic corneas that appear to be related to cell adhesion and basement membrane alterations, decreased innervation, and poor wound healing [44-46]. Using adenoviral gene therapy with overexpression of c-met that is downregulated in diabetic corneas [47], we were able to bring basement membrane protein patterns and wound healing times in organ-cultured diabetic corneas close to normal [30]. Other possible ways to normalize these corneas could be a restoration of normal signaling of the epidermal growth factor receptor (EGFR) axis that is downregulated by high glucose and diabetes [48,49], silencing of specific proteinases [31] or a blockade of opioid growth factor - opioid growth factor receptor system with naltrexone [50].

Mechanisms responsible for the epithelial changes in diabetic corneas are still not well understood. One possibility is that epithelial alterations could be triggered or exacerbated by abnormal innervation, which may be the case with ulcers [51]. However, several lines of evidence support direct detrimental action of hyperglycemia in diabetes on corneal epithelium. In diabetic rabbits, corneal wound healing is not delayed [52], although corneal neuropathy develops [53]. In human corneal organ culture where corneas are denervated, delayed diabetic epithelial wound healing persists [30]. In normal organ-cultured porcine corneas, high glucose causes delayed epithelial wound healing [48]. Because LESC and their immediate progeny (TA cells) play a key role in the epithelial maintenance and renewal, these data support the hypothesis that LESC and or/TA cells may be altered in the course of diabetes. Using a large panel of antibodies to putative LESC/limbal basal epithelial markers we tested this hypothesis using ex vivo human corneas, as well as organ-cultured diabetic corneas upon viral-induced c-met overexpression.

In the ex vivo corneas, many tested markers including keratins 15, 17 and 19, as well as Bcrp1/ABCG2, ΔNp63α isoform, N-cadherin, laminin γ3 chain, and β_1_ integrin were significantly downregulated in diabetic compared to normal limbus. In some cases, such as with integrin β_1_ or laminin γ3 chain, the immunostaining intensity was diminished. In other cases, such as with K17, ABCG2, or N-cadherin, the number of positive cells was markedly reduced in the diabetic limbus. This is the first demonstration of changes in LESC marker expression in a common disease that does not involve LESC deficiency. It may be suggested that the observed differences in marker expression between normal and diabetic corneal limbus relate to functional abnormalities of stem cell niche in diabetes. At present one can only speculate on what kind of dysfunction such reduced marker expression would be related. A plausible candidate would be impaired cell migration translating into slower and incomplete wound healing in diabetic corneas. The data support the idea that stem cell niche alterations may underlie poor wound healing and other epithelial abnormalities typical for diabetic corneas. It would also be important to understand whether diabetes changes marker expression in LESC, TA cells or both. The generally even distribution of most studied putative LESC markers in the limbal basal cells (comprised by LESC and TA cells) would favor the hypothesis that reduced expression of these markers in diabetic corneas is applicable to both LESC and TA cells.

It was interesting to examine whether gene therapy that can bring diabetic corneas closer to normal in terms of specific protein expression and wound healing rates would also change the expression of putative LESC markers in diabetic corneas toward normal patterns. To this end, we used organ-cultured diabetic corneas following gene therapy with *c-met,* which had significantly improved epithelial wound healing and expression of basement membrane markers and signaling intermediates [30].

Compared to vector treatment, *c-met* treated corneas displayed enhanced staining for several putative LESC markers, which became similar to normal ex vivo limbus. These data attest to the feasibility of using specific gene therapy to normalize the functions of LESC in diabetic corneas, which may be useful for LESC transplantation in diabetics. However, not all the markers altered in diabetic ex vivo corneas showed increased staining upon *c-met* gene transduction (e.g., β_1_ integrin) suggesting that one-gene therapy was not enough for corneal normalization.

A partial effect of c-met upregulation on LESC marker expression could be related to the influence of this gene therapy only on certain cell signaling pathways. As we showed before [30], c-met overexpression in diabetic corneas causes normalization of epithelial wound healing by restoring signaling through p38. However, overexpression of proteinases cathepsin F and matrix metalloproteinase-10 (MMP-10) in diabetic corneas or incubation of normal corneas in high glucose appear to reduce migration-promoting EGFR signaling through Akt phosphorylation [31,48]. Our preliminary data showed that a combined gene therapy with c-met overexpression and shRNA silencing of cathepsin F and MMP-10 brought diabetic corneas significantly closer to normal in terms of epithelial protein expression, p38 and Akt phosphorylation, and wound healing time than c-met upregulation alone. Therefore, by a concerted acting on several key signaling pathways, this combination could possibly exert a greater positive effect on putative LESC marker expression in the diabetic limbus.

Based on limbal location, little or no expression in the central cornea, preferential expression in basal limbal epithelial cells, various putative LESC markers have been proposed, such as K8, K15, K17, K19, Bcrp1/ABCG2, ΔNp63α, N-cadherin, laminin γ3 chain, β_1_ integrin, TCF4, frizzled 7, SOD2, epiregulin, Notch-1, α-enolase, vimentin, C/EBPδ, SPON1, and nectin-3 [23,32,33,35,36,41,54-65]. However, despite numerous attempts, no single and reliable LESC marker has been identified so far. This is in part due to the fact that unambiguous identification of LESC has been difficult. These cells are generally considered as largely non-proliferating, or slow cycling. Based on this criterion many authors agree that if corneal cultures or animal corneas in vivo are labeled with tritiated thymidine or bromodeoxyuridine and then chased for a while (at least several weeks), the few corneal cells that retain the label should be considered LESC. This promising strategy has been used to examine which markers are expressed by these cells. They were found to stain for K14, K15, CDH3 (P-cadherin), Wnt-4 [61], as well as to contain high levels of integrins β_1_ and β_4_ [60]. However, these markers are expressed not only in putative LESC but also in other limbal cells, as well as in central corneal cells (e.g., both integrins) and thus cannot be considered specific for LESC. Additional experiments with label-retaining cells using a large panel of antibodies are definitely needed to establish which existing markers are more specific for LESC. Currently, it is generally agreed that a combination of several markers should be used to characterize the presence of LESC in tissues and cultures.

The problem of LESC markers has gained wide attention because of recent success in transplantation of cultured limbal epithelium to patients with LESC deficiency [26,66-71]. Unfortunately, not all such cultures, especially when only small amounts of biopsied tissue were available for autologous transplantation, have been characterized as to the expression of putative LESC markers. Some authors, however, to standardize the cultures for successful transplantation, did examine one to several markers, e.g., p63 and K19 [69,70], confirming the presence of LESC-like cells in the transplanted cultures. In line with low content of LESC in corneal tissue, successful transplantations could be achieved when the fraction of p63-positive cells exceeded 3% [70].

In summary, we provide here the first account of significant alterations of limbal stem cell compartment in human diabetic corneas with respect to several commonly used putative LESC markers. These abnormalities may lead to diabetic LESC dysfunction and to clinically observed epithelial problems in diabetics including poor wound healing. Partial normalization of these pathological changes by c-met overexpression may offer a possibility of improving LESC function and general corneal health in diabetes by specific gene therapy. Another promising approach could be autologous transplantation of limbal epithelial cells to diabetic patients with advanced disease after prior normalization of their marker expression levels by gene therapy during culture.
